# Enhancing Pragmatic Communication in Healthy Aging: A Preliminary Investigation of the Long-Term Effects of the Advanced Cognitive Pragmatic Treatment

**DOI:** 10.1007/s10936-026-10291-4

**Published:** 2026-08-21

**Authors:** Ilaria Gabbatore, Giorgia Vegna, Dize Hilviu, Ariele Merlini, Francesca M. Bosco

**Affiliations:** 1 Department of Humanities, University of Turin, Turin, Italy; 2 GIPSI - research Group on Inferential Processes in Social Interaction, University of Turin, Turin, Italy; 3 Department of Psychology, University of Turin, Turin, Italy; 4 Neuroscience Institute of Turin, University of Turin, Turin, Italy

**Keywords:** Healthy aging, Pragmatic ability, Enhancement, Comprehension, Production

## Abstract

The aging process is associated with changes in cognitive and communicative skills, including pragmatic one, the ability to appropriately use language and other expressive means in social contexts. Despite its importance for quality of life and autonomy, the decline of pragmatic ability has received little attention in aging research and intervention. This preliminary study aims to investigate the efficacy and specificity of the Advanced Cognitive Pragmatic Treatment (Advanced-CPT), a novel training program designed to improve pragmatic performance in healthy older adults by targeting different expressive modalities, i.e., linguistic, extralinguistic, and paralinguistic, in addition to sensitivity to context. Twenty-four healthy participants aged 65–86 years completed the nine-week group training. Participants were assessed immediately before and after the training, and at a three-month follow-up. Cognitive functions, i.e., attention, memory, processing speed, executive functions and Theory of Mind (ToM) were also evaluated pre- and post-training. Participants showed a significant improvement in overall pragmatic performance from pre- to post-training, which was maintained at follow-up. Significant improvements were noted in both pragmatic comprehension and production, and in both linguistic and extralinguistic expressive means. A trend towards enhancement was also observed in paralinguistic ability, although the changes did not reach statistical significance, while no significant change emerged in sensitivity to the context. As expected, cognitive and ToM measures remained stable. The findings provide preliminary support for the potential of the Advanced-CPT to improve pragmatic ability in healthy aging, with gains that remained detectable at follow-up. Cognitive and ToM measures did not show comparable pre-post changes, suggesting that the main improvements observed in the present study were confined to the pragmatic domain.

**Supplementary Information:**

The online version contains supplementary material available at https://doi.org/10.1007/s10936-026-10291-4.

## Introduction

In recent decades, demographic development throughout Europe has shown an overall trend towards an increasingly aging population. Among European countries, Italy has the highest percentage of older adults, and according to the European Commission’s latest Ageing Report (European Commission, 2024), this percentage will continue to rise in the coming decades. The progressive increase in life expectancy has led to a parallel interest in ensuring not only a longer life, but also a life of sustained quality. In this context, the aging population has become a key issue for research and policies. Aging has long been associated with general cognitive decline (Glisky, 2007; Salthouse, 2010): as people age, they may experience increasing difficulties due to both functional and structural changes (Fjell et al., 2017) in various cognitive domains, such as memory (Nyberg et al., 2012), attention (Godefroy et al., 2010), and executive functions (EFs), a set of cognitive abilities such as planning, working memory, inhibition, and shifting that support goal-directed behavior (Diamond, 2013; Jurado & Rosselli, 2007; Klencklen et al., 2017; Miyake et al., 2000). Linguistic functions are also influenced by age, especially in terms of fluency. Singh-Manoux et al. (2012) found that both phonemic and semantic fluency, assessed by asking individuals to generate words that begin with a specific letter or belong to a specific category, begin to decline in midlife (45–50 years of age) and may have an increasing impact on everyday functioning. These results suggest a general age-related decline in the ability to efficiently access and recall verbal information (Singh-Manoux et al., 2012). By contrast, certain aspects of language, such as semantic knowledge, appear to remain relatively stable in older adulthood and may even be preserved in the early stages of cognitive impairment (Harada et al., 2013).

Some studies (Charlton et al., 2009; Duval et al., 2011a; Merlini et al., 2026) indicate that executive functioning is linked to Theory of Mind (ToM) (Premack & Woodruff, 1978), the ability to attribute mental states to oneself and others, suggesting that age-related difficulties in ToM may be at least partly due to a decline in underlying cognitive control processes. Indeed, such ability has also been shown to decline with age (Bottiroli et al., 2016; Henry et al., 2013; Raimo et al., 2022). ToM is a multidimensional construct (Bosco et al., 2009) that includes a cognitive (i.e., understanding of others’ knowledge, intentions, and beliefs) and an affective component (i.e., processing of others’ emotions and feelings) (Shamay-Tsoory & Aharon-Peretz, 2007). Notably, the above-mentioned decline appears to be selectively associated with the cognitive component of ToM, rather than with its affective aspects (Bottiroli et al., 2016). While Bottiroli et al. (2016) proposed that the age-related reduction in working memory updating plays a mediating role in this selective decline, other studies find no relation between cognitive ToM and executive task performance in aging (Cavallini et al., 2013; Duval et al., 2011b). Overall, the evidence remains mixed, suggesting that while ToM and EFs may be related to some extent, they are likely to involve at least partially distinct processes.

Despite the extensive attention paid to cognitive decline, comparatively less attention has been devoted to pragmatic ability in the context of healthy aging. Traditionally, pragmatics is defined as the ability to use and interpret language appropriately in social contexts (Levinson, 1983). More recently, it has been pointed out that pragmatic competence goes well beyond language alone. As discussed by Bara (2010) and by Holler and Levinson (2019), it also involves extralinguistic means, i.e., gestures, facial expressions and proxemics, and paralinguistic cues, i.e., tone of voice and intonation. Available research suggests that pragmatic ability is not immune to age-related changes, as a decline has been observed in both the production and comprehension of pragmatic content (Baraldi & Domaneschi, 2024; Hilviu et al., 2022; Marini et al., 2005; Petriglia et al., 2025), although it is relatively under-researched and the literature has mainly focused on linguistic expressive means. In terms of pragmatic production, older adults tend to produce more verbose and less informative utterances compared to younger speakers, indicating difficulties in maintaining conversational coherence, relevance, and efficiency (Arbuckle & Gold, 1993; Hilviu et al., 2025). These challenges suggest a reduced ability to adapt language use to the social context and conversational goals, adhering to both conversational norms and situational appropriateness (Juncos-Rabadán et al., 2005). Regarding comprehension, prior research has shown that older adults have difficulty understanding contextually nuanced language, e.g., humor (Bischetti et al., 2023), and figurative expressions such as metaphors and proverbs (Morrone et al., 2010; Sundaray et al., 2018; Uekermann et al., 2008).

Observable age-related changes go beyond language itself. Ageing also affects the extralinguistic component, with older adults showing reduced efficiency in understanding and producing gestures and facial expressions (Schubotz et al., 2021; Theocharopoulou et al., 2015). For example, Isaacowitz and Stanley (2011) reported that older adults tend to rely more on verbal content and ignore gestural cues when both are present, suggesting a shift in the integration of multimodal information. Moreover, the ability to interpret or use paralinguistic cues such as emotional prosody and intonation has been found to decline with age (Mitchell, 2007; Ross & Monnot, 2011). For example, older adults have difficulty grasping emotional tones, which can affect their understanding of the speaker’s intention or attitude.

Finally, when examining pragmatic ability, it is also important to consider the individual’s capacity to adapt communication to the social context, adhering to both conversational norms and situational appropriateness. Research has shown that certain aspects of this ability can change during the aging process. There is, indeed, evidence that the ability to recognize which communicative behaviors are socially appropriate in a given context declines with age (Halberstadt et al., 2011). Moreover, older adults often tend to be more verbose and less informative than younger people (Marini et al., 2005; Juncos-Rabadán et al., 2005).

These findings highlight the need for an accurate assessment of both pragmatic comprehension and production in healthy aging emerged: Hilviu et al. (2022) conducted a comprehensive assessment of communicative-pragmatic competence in three age groups: young adults (20–40 years), older adults (65–75 years) and senior-older adults (76–86 years). The study found that extralinguistic skill was most affected by aging. Both groups of older adults performed significantly worse on tasks with nonverbal cues than younger adults. Interestingly, the difference in performance between the two older groups was not significant, suggesting that the deterioration in this area may occur earlier in the aging process and then stabilize. The ability to comprehend and produce paralinguistic cues also deteriorated with age. Older adults were found to have particular difficulty understanding affective prosody, even in the absence of hearing impairment. In contrast, linguistic and conversational abilities appeared to be relatively well preserved across all age groups. By contrast, performance on the contextual scale, which measures the appropriateness of communicative behavior in social contexts, declined significantly only in the oldest age group, suggesting that the ability to modulate language and behavior according to social norms may remain stable into later stages of aging. Overall, these results point to the importance of taking a comprehensive approach when assessing pragmatic ability in healthy aging, as it encompasses a variety of expressive means and components that may be differentially affected by the aging process. This issue is particularly relevant in the Italian context, where recent research has provided increasingly fine-grained tools and evidence on pragmatic, narrative, and discourse functioning across adulthood and older age, indicating that age-related communicative changes are not limited to isolated pragmatic phenomena, but also involve broader discourse-level abilities. In healthy aging, pragmatic decline appears to be intertwined with narrative performance (Petriglia et al., 2025), with changes emerging in informativeness, discourse organization, and the ability to manage subjectivity and emotion expression during storytelling (Gallo et al., 2025). These findings reinforce the need for a multidimensional assessment of communication in older adults, capable of capturing pragmatic functioning together with narrative and discourse production skills (Marini et al., 2025).

It has been suggested that the overall level of cognitive ability might predict pragmatic performance (Daniluk & Borkowska, 2020). Recent evidence also suggests that pragmatic performance is associated with broader cognitive functioning. In the Italian context, studies on the APACS Brief (Bischetti et al., 2025) and APACS Brief Remote (Bischetti et al., 2024) have shown that pragmatic ability, particularly in discourse and non-literal language, is meaningfully related to cognitive substrates, supporting the view that overall cognitive efficiency may contribute to communicative-pragmatic performance. Specifically, pragmatic ability interacts with ToM and other cognitive functions such as EFs, also in aging (Parola et al., 2026), but the nature and direction of their relationship are still not clear and widely debated in the current literature (Bosco et al., 2018; Frau et al., 2024). As for EFs, skills such as working memory, inhibition, and flexibility appear involved in various pragmatic tasks in aging (Bambini et al., 2021). Similarly, ToM has also been shown to be involved in the ability to narrate (Hilviu et al., 2025), as well as in the comprehension of mental metaphors (Ceccato et al., 2025) and humor (Bischetti et al., 2023). Overall, cognitive functioning and ToM appear to be partially associated with pragmatic ability, but the available data suggest that these domains are not fully overlapping and pragmatic ability is not explained as a compound of these other cognitive abilities (Babarczy et al., 2024; Bambini et al., 2016; Bosco et al., 2018a, b, c, 2019; Kissine, 2021).

Quite surprisingly, despite the impact of communicative ability on personal autonomy and quality of life (Messer, 2015), there has been limited research on the improvement of pragmatic ability in healthy aging. To the best of our knowledge, only one study has systematically investigated the potential for enhancing pragmatic ability in healthy older people. Bambini et al. (2020) analysed the efficacy of a structured training program, PRAGMACOM, which focused on both comprehension and production of linguistic communicative acts. The training program included activities designed to stimulate pragmatic skill, including the ability to recognize implicit meanings, navigate social norms, and adjust communicative strategies based on feedback from the social environment. Through the intervention, participants showed significant improvements in their ability to interpret and express communicative intentions beyond the literal meaning. The results of this study provided convincing evidence that, at least with respect to linguistic means, older adults’ pragmatic competence can be improved. However, given the multifaceted nature of pragmatic competence, any attempt to improve it in older adults must equally embrace a comprehensive approach. This includes not only strengthening core linguistic skills, but also reinforcing paralinguistic and extralinguistic means, in addition to the ability to adapt communicative behavior to social norms and contexts. The ability to navigate social appropriateness plays a crucial role in daily interactions and contributes to emotional well-being, social integration and overall quality of life (Messer, 2015). Therefore, training programs should aim to address all the different components of pragmatic ability to promote meaningful improvements in real-life interactions. Isolated interventions aimed at individual expressive means, such as language comprehension or gesture recognition, may fall short if they do not address the dynamic interplay of these interrelated cues. The study of Bambini and colleagues (2020) on PRAGMACOM also raised important questions about the relationship between pragmatic ability and other cognitive functions. Interestingly, individuals in the control group who underwent cognitive training (targeting memory, speed of processing, and reasoning skills) showed improvement in the communicative domain similar to that observed in the group who took part in the PRAGMACOM training. These results highlighted the need to further clarify how pragmatic skill is intertwined with processes such as memory, ToM and EFs.

To this end, the present study aims to present and provide a preliminarily examination of the effects of participation in the Advanced Cognitive Pragmatic Treatment (Advanced-CPT), a novel Italian intervention based on Cognitive Pragmatic Theory (Bara, 2010). This theoretical framework highlights the inferential processes underlying human communication (see also Bosco et al., 2004; Bosco & Bucciarelli, 2008) and enabling communicators to go beyond literal language and capture intended meanings. According to the theory, a communicative act may be expressed through various expressive modalities, such as speech, gestures, body movements, and facial expressions, all of which should be seen as alternative ways of manifesting the same communicative competence. A key aspect of the theory is that communication is understood as a process involving several distinct inferential steps of interpretation. Beginning with the literal meaning and moving through the understanding of the speaker’s intended meaning, this process enables the interlocutor to grasp the communicative effect the speaker aims to achieve (see also Airenti et al., 1993).

The Advanced-CPT shares its theoretical basis with the Cognitive Pragmatic Treatment (CPT; Gabbatore et al., 2015). The original version of the CPT consisted of small-group sessions held twice a week, targeting key aspects of pragmatic communication. Originally developed for neurological (Gabbatore et al., 2015) and psychiatric populations (Bosco et al., 2016), it focused on linguistic, extralinguistic, and paralinguistic skills, as well as social appropriateness, conversation, self-awareness, social abilities, and planning. In an ecological setting, participants practiced communication strategies for everyday situations, supported by therapist feedback and self-monitoring. Grounded in the idea that communication relies on creating and sharing meaning across different expressive modalities, the program aimed to help participants go beyond literal meanings and understand communicative intent. In its original version, the CPT has proven effective with individuals with brain injury (TBI; Bosco et al., 2018a, b, c; Gabbatore et al., 2015; Parola et al., 2019), schizophrenia (Bosco et al., 2016) and in autism (Gabbatore et al., 2022; Hilviu et al., 2023), proving also effective in determining neuronal changes, as assessed by resting state functional connectivity (Gabbatore Bosco et al., 2017; Sacco et al., 2016), besides behavioral improvements.

Like the CPT, the Advanced-CPT targets the inferential dimension of communication and the ability to integrate different expressive modalities such as spoken language, tone of voice, gestures, and facial expressions. However, it is noteworthy that the Advanced-CPT is a newly developed program and includes important innovations: it has been developed specifically for healthy older adults and encompasses new materials and multimedia resources to better reflect contemporary communicative contexts. A key feature is its focus on intergenerational communication, which aims to improve the quality of everyday interactions between older adults and members of younger generations.

### Aims of the Study

This preliminary study was designed to test two main hypotheses. First (Hp1), we hypothesized that participation in the Advanced-CPT would lead to significant improvements in pragmatic ability in healthy older adults; moreover, we expected these changes to be maintained over time, and remain observable at a three-monthfollow-up. More specifically, we expected these improvements (Hp1a) to involve both pragmatic comprehension and production, and (Hp1b) to extend beyond the linguistic dimension to include other expressive modalities, namely, extralinguistic and paralinguistic means in addition to sensitivity to context. Second (Hp2), we expected the improvement over time to emerge only in pragmatic competence, the target variable of the program, whereas no comparable changes were expected in the cognitive and ToM measures included as control tasks.

## Methods

### Participants

Twenty-five individuals volunteered to participate in the study, but one of them had to withdraw due to medical reasons, which brought the final sample to 24 participants (10 men). The age range was 65–86 years (*M* = 72.92; *SD* = 6.26), with an educational level of 9–20 years (*M* = 13.79; *SD* = 2.59). To ensure that there were no severe cognitive or language impairments, eligibility was determined based on performance above established cut-offs on the Token Test (≥ 4.5) (Spinnler & Tognoni, 1987), the Naming Scale of the Aachen Aphasia Test (≥ 108/120) (AAT; Huber et al., 1982), and the Montreal Cognitive Assessment (MoCA; Nasreddine et al., 2005), with a cut-off of 19.5 according to Italian norms (Conti et al., 2015). Participants were enrolled in the study if Italian native speakers; those with a history of psychiatric or neurological disorders, significant sensory impairment (vision or hearing), prolonged substance abuse, or treatment with mood stabilizers were excluded.

### Procedure

Participants were recruited with the support of two local associations in the Torino area: *Università delle Tre Età (Nichelino, Turin)*, an educational institution open to people of all ages and educational backgrounds, promoting lifelong learning opportunities, and *Associazione Solidarietà Volontariato a Domicilio Onlus (Turin)*, which supports families of people with dementia and neurodegenerative disorders, helping them to reduce isolation and providing emotional assistance. Additionally, participants were involved through the distribution of flyers in different areas of the province of Torino, through referrals among friends and acquaintances and as a result of public engagement activities carried out by the research group. The study was approved by the Bio-Ethical Committee of the University of Turin (protocol n. 0193556) and was conducted in accordance with the Helsinki Declaration. Prior to the study, all participants were informed about the aims of the study and they gave informed written consent. They were also informed that their participation was voluntary, and they could withdraw at any time.

Participants were enrolled in a single-group pre-test/post-test quasi-experimental study with a 3-month follow-up. No control group was included at this stage, as the primary aim of this preliminary investigation was to pilot the Advanced-CPT protocol, assess its feasibility and acceptability, and evaluate the sensitivity of the selected outcome measures before conducting a larger controlled trial. Participants were assessed at three time points to track changes over time: before the training (T0), immediately after its completion (T1), and in a follow-up three months later (T2). The assessments at T0 and T1 consisted of three individual sessions, in which screening tests, cognitive abilities, EFs, ToM, and pragmatic ability were assessed. The follow-up assessment at T2, conducted in a single individual session, focused exclusively on pragmatic ability to assess the long-term stability of any observed changes.

**Fig. 1:**
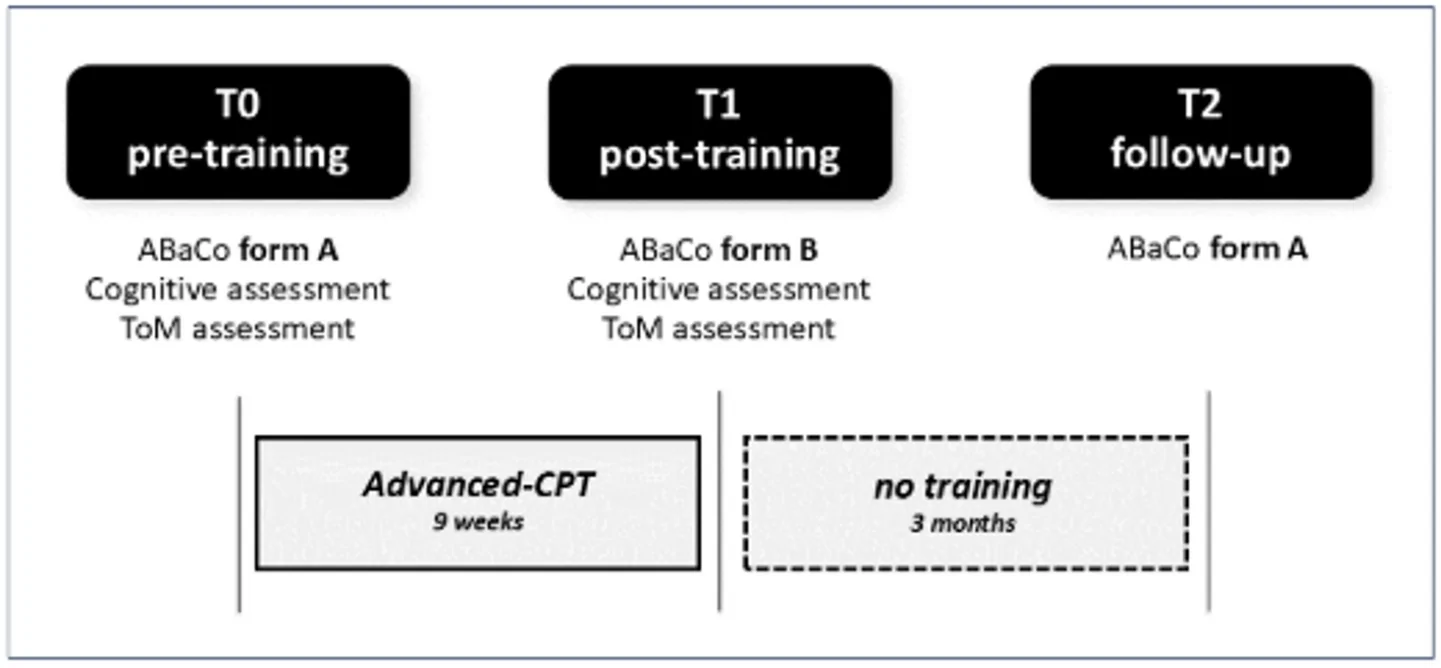
Quasi-experimental design and procedure

The one-group pre-test/post-test quasi-experimental design is illustrated in Fig. 1.

### Advanced Cognitive Pragmatic Treatment

The Advanced-CPT was designed to enhance communicative-pragmatic ability by targeting its different components and expressive means. The Advanced-CPT is grounded in the theoretical framework of Cognitive Pragmatic Theory (Bara, 2010), which conceptualizes communication as an inferential process that is realized by different expressive means; the training translates these theoretical principles into a structured and supportive environment in which participants can practice ecologically valid tasks that reflect real-life communicative challenges. In particular, the Advanced-CPT draws on the theory’s emphasis on the inferential effort required to process different pragmatic phenomena such as sincere communicative acts (in which the speaker’s intent matches literal meaning), ironic acts (in which intended meaning opposes the literal one) and deceptive communicative acts (in which the spoken utterances do not align with the shared knowledge between the interlocutors) each demanding increasing levels of inferential load. It aims to strengthen this inferential ability, allowing individuals to move beyond the literal meaning of utterances and grasp implicit messages, conversational nuances and social cues. Moreover, the program promotes the integration of multiple expressive modalities, encouraging participants to refine not only verbal communication but also their use of prosody, gestures and facial expressions to correctly interpret and express communicative intentions, thus promoting a more natural and effective interaction style. Furthermore, the training is based on the principle that increasing self-awareness of one’s own communication difficulties is crucial for meaningful and lasting improvement. Participants are encouraged to monitor and reflect on their own communication processes in order to develop a more conscious and adaptive approach to social interactions, in line with the theory’s attention to intentionality and mental state attribution in social exchanges.

The training incorporates progressively complex activities specifically designed to be stimulating even for healthy older adults, to ensure engagement. Comprehension exercises include scenes from movies or short films that provide rich and natural communicative contexts. Role-playing activities are structured to reflect real-life situations; they are designed to involve multiple participants simultaneously, thus reinforcing the social and collaborative dimension of the exercises. Similarly, prosodic activities also follow a multimodal approach that encourages the fluid integration of different communicative components such as vocal tone, facial expressions, and body language to improve expressiveness and communicative effectiveness.

**Fig. 2:**
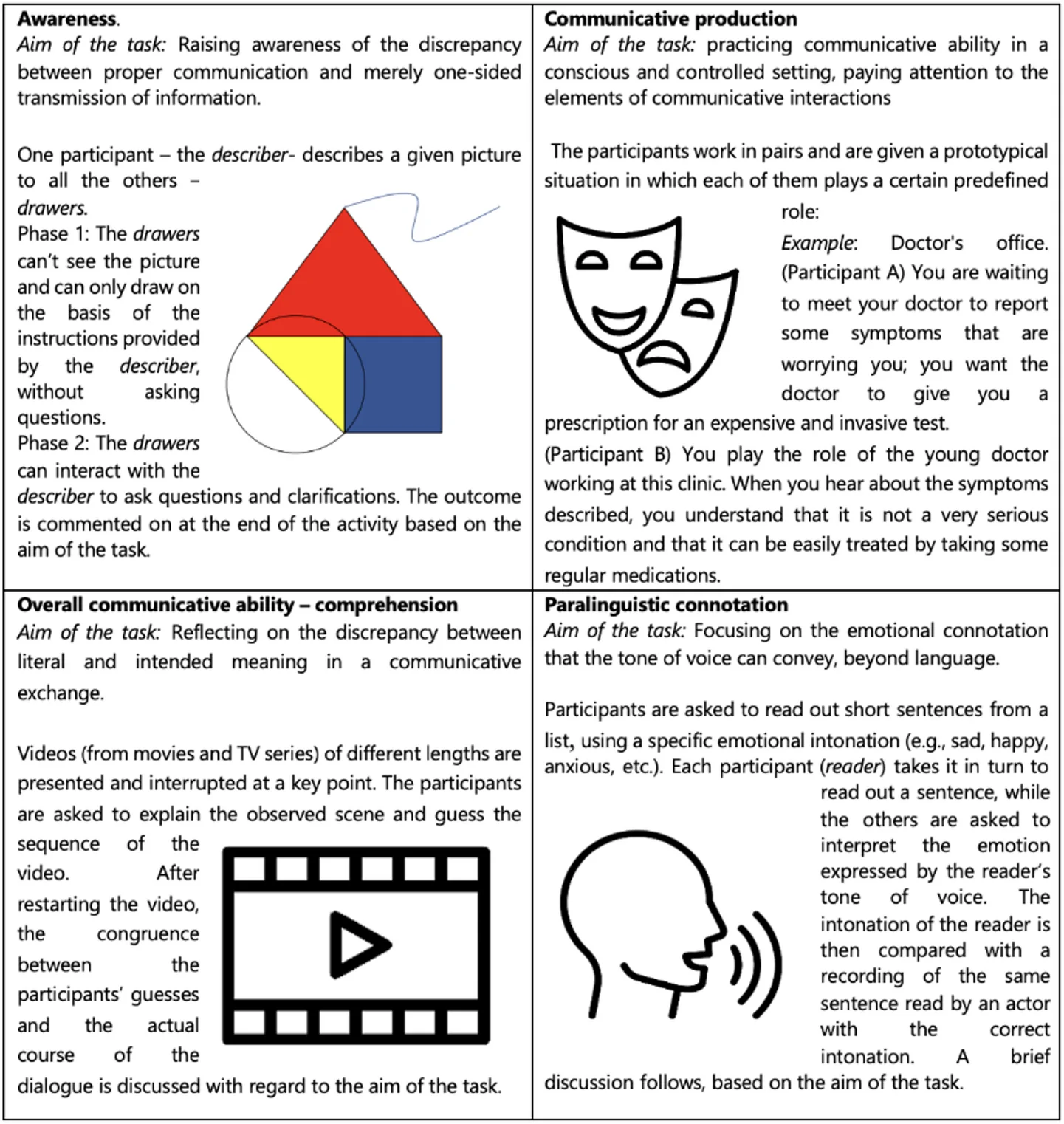
Selection of task types composing the advanced cognitive pragmatic training

Another innovative aspect of the Advanced-CPT is the inclusion of activities specifically geared towards intergenerational communication. They target the ability to draw inferences from idiomatic expressions, metaphors and colloquialisms commonly used in contemporary language. Misunderstandings in these areas often hinder fluent conversation for older adults, so this training is particularly valuable in promoting more effective and confident interactions. Additionally, the program includes exercises aimed at improving communicative appropriateness when using new digital communication tools such as email, instant messaging and social media. Figure 2 reports a small selection of tasks composing the Advanced-CPT.

The training is structured into nine weekly sessions, each lasting approximately 90 min with a 10-minute break, conducted in small groups of 5 to 6 participants. The sessions were led by eight different master’s students, with a bachelor’s in Psychology, under the supervision of experienced psychologists and psychotherapists with expertise in psychology of communication and pragmatics. Each session is structured around dedicated modules that address the above-mentioned specific aspects of communication. A summary of the topics covered in each session is provided in Table 1.

**Table 1:** Overview of the advanced cognitive pragmatic treatment’s topics

Session		Key content
1	Initial Presentation and Awareness	Participants are invited to reflect on their motivations and expectations for the program. The session introduces communication as a collaborative process rather than a simple transfer of information, emphasizing the integration of linguistic and extralinguistic elements.
2	Fundamental Communicative Skills	Focus on communicative intent and the importance of interpreting meaning beyond verbal content. Participants learn to consider extralinguistic (i.e., gestures, posture, tone of voice) and contextual cues (shared background knowledge) for deeper understanding.
3	Extralinguistic Skill	Highlights the role of nonverbal communication, such as facial expressions and gestures, in conveying meaning. Participants develop awareness of how nonverbal signals provide critical information about a speaker’s emotions and communicative intent.
4	Paralinguistic Skill	Explores how tone, intonation, and prosody shape communication. Participants reflect on the ways these elements contribute to meaning, emotion, and social interpretation.
5	Cognitive Flexibility in Communication	Emphasizes the ability to adapt communication based on the listener’s needs and feedback. Participants practice producing contributions that are clear, relevant, and appropriately detailed.
6	Clarity and Effectiveness in Expression	Reinforces the need to adjust communication based on the interlocutor and context. Participants work on structuring messages that are concise, relevant, and easily understood.
7	Narrative Skills	Focuses on distinguishing between relevant and irrelevant information for effective storytelling. Participants practice summarizing key points while reducing verbosity.
8	Social Appropriateness and Digital Communication	Encourages reflection on how communication tools and language registers should align with social context and message content. Participants discuss how to adjust tone and style in different social and digital interactions.
9	Final Session and Self-Reflection	Participants review key concepts from the program and analyze personal improvements using video excerpts from previous sessions. The course concludes with a discussion on progress, final reflections, and participant feedback.

Throughout the sessions, self-monitoring and feedback from the facilitator and peers, play a crucial role in reinforcing progress and deepening participants’ understanding of their own communicative behaviors and those of others. Each training session is organized as follows:

*Introduction and overview* Each session begins with a brief recap of previously covered concepts, followed by an introduction to the day’s topic. Participants are encouraged to reflect on the relevance of these concepts in their daily lives.

*Comprehension* The first part of the session focuses on comprehension. Participants engage in a task related to the session’s theme, using multimedia materials and interactive exercises. For instance, they watch video clips in which actors communicate using different modalities (e.g., gestures in extralinguistic sessions) and are then asked to analyze and discuss the interactions they observed.

*Production* The second part of the session focuses on production. In most sessions, participants take part in role-playing activities that correspond to the communicative modality or specific aspect of communication being addressed in that specific session. Each participant is assigned a character with predefined traits and a scenario. They must step into their role and navigate a social conflict while staying true to their character’s personality.

*Conclusion and homework* Each week, participants are encouraged to apply the training concepts in their everyday interactions and recall relevant experiences to discuss in the following session.

All sessions are video-recorded, with participants’ consent, to provide an objective and analytical tool for reviewing session content. Video feedback, both during and at the end of the program, plays a crucial role in helping participants develop greater awareness of their difficulties and progress over time. A full description of the Advanced-CPT is provided in Supplementary Material.

### Materials

#### Communicative-Pragmatic Assessment

**Fig. 3:**
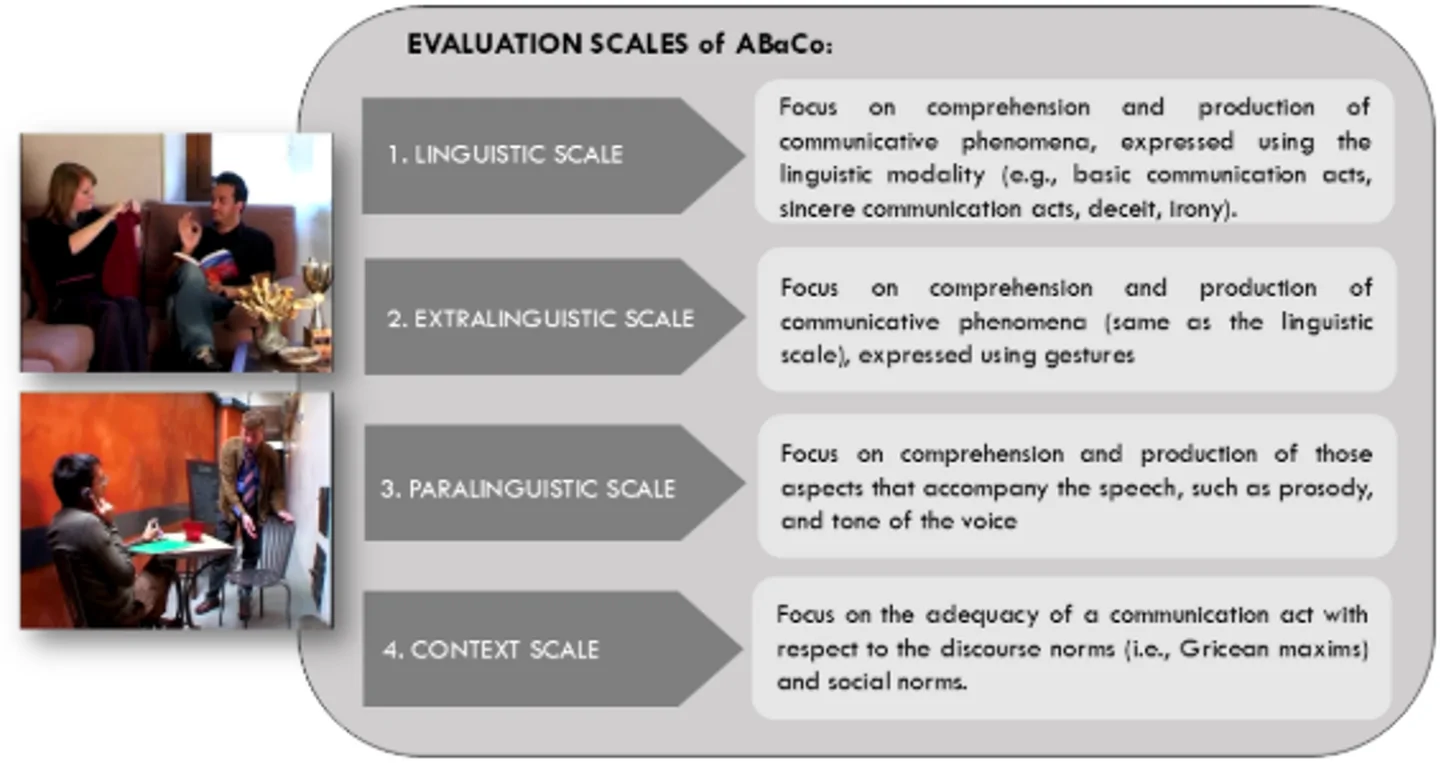
Brief description of the ABaCo’s scales

Communicative-pragmatic ability was assessed using the Equivalent Forms (Bosco et al., 2012) of the Assessment Battery for Communication (ABaCo; Angeleri et al., 2015; Angeleri et al., 2026) a standardized tool designed to measure a broad range of pragmatic phenomena via various expressive modalities, including verbal language, gestures, and prosody, in both comprehension and production. Equivalent forms are different versions of the same tool, focusing on the same pragmatic phenomena and characterized by the same level of difficulty. To reliably track changes over time while minimizing the risk of learning effects in case of test-retest, both versions of the ABaCo were administered. Form A was used for the pre-training (T0) and follow-up (T2) assessments, whereas Form B was employed at post-training (T1). The two versions assess the same pragmatic domains through identical task structures but with different items, ensuring that any improvement reflects genuine skill development rather than familiarity with test content. Each version includes 68 items distributed across four scales - linguistic, extralinguistic, paralinguistic, and contextual - with a total administration time of approximately 45 min. Each scale assesses pragmatic phenomena in comprehension and production. See Fig. 3 for a description of ABaCo’s scales.

The assessment involves both live interactions with the examiner and brief video-based tasks, where participants are required to interpret the actors’ communicative intent or produce appropriate responses for the represented situation. Each session is recorded on video for offline coding by an independent judge, who was unaware of the aim of the study. Every item is rated 1 if correct or 0 if incorrect. For each participant, scores were averaged across items within each scale and for production, comprehension and ABaCo composite total scores, resulting in a proportion of correct responses ranging from 0 (no correct responses) to 1 (all responses correct). ABaCo has demonstrated robust psychometric properties, including high internal consistency within each scale (0.63 ≤ *α* ≤ 0.91), strong inter-rater reliability (0.76 ≤ *κ ≤* 0.96), and well-established content and construct validity (Bosco et al., 2012; Angeleri, 2015).

#### Cognitive Assessment

A set of neuropsychological tests was conducted before and after the training to assess a range of cognitive functions. For this part of the assessment, administration and scoring procedures were conducted by the same person.

*Attention.* To assess selective and alternating attention, we administered the Trail Making Test (TMT; Reitan, 1958) form A and B. TMT A measures visual selective attention by requiring participants to sequentially connect numbered circles as quickly as possible. TMT B assesses cognitive flexibility and task-switching ability, as participants must alternate between numbers and letters in ascending order. The TMT has shown good test-retest reliability, with Spearman’s coefficients 0.70 ≤ *r*_*s*_ ≤ 0.81; moreover, its construct validity is supported by significant associations with global cognitive functioning and executive-attentional measures (Siciliano et al., 2019).

*Memory.* To evaluate short-term memory, we used the Digit Span Test – Forward (Wechsler, 2008), a task that measures auditory short-term memory by requiring participants to recall a sequence of digits in the presented order. The test had been selected for its psychometric properties, with adequate test–retest reliability in the WAIS-IV standardization sample (*r* = 0.77; Wechsler, 2008) and good internal consistency (*α* = 0.81; Gignac et al., 2019). For short- and long-term memory, we used the Immediate and Deferred Story Recall (Spinnler & Tognoni, 1987). In this task, participants listen to a short story and are asked to recall its details immediately and after a delay, allowing the assessment of both immediate retention and the consolidation of information over time. Italian normative and clinical validity data are available for the short-story recall test, supporting its use in the assessment of episodic verbal memory (Capitani et al., 1994; Carlesimo et al., 2002).

*Processing speed.* The first two parts of Stroop Color Word Test (SCWT; Stroop, 1935) were employed to assess processing speed. In the first part, participants must read a list of words corresponding to the names of certain colors as quickly as possible. In the second part, they are asked to name the colors displayed. Stroop WT and CT are measures of time, while WI and CI are given by the number of items correctly named in the first 30 s. Psychometric evidence supports the use of the SCWT, which has shown adequate test-retest reliability with Spearman’s coefficients 0.671 ≤ *r*_*s*_ ≤ 0.831 (Franzen et al., 1987), while its construct validity is supported by evidence showing that the direct and interference scores reflect theoretically meaningful components such as visual search speed, working memory, and conflict monitoring (Periáñez et al., 2021).

*Executive Functions.* We selected key executive functions based on Miyake and colleagues’ (2000) model (i.e., working memory updating, cognitive flexibility, and inhibition) with the addition of strategic planning to obtain a comprehensive assessment. To measure *working memory*, we used the Digit Span Test – Backward (Wechsler, 2008), a task that requires participants to recall a sequence of digits in reverse order. To assess *strategic planning* and *problem-solving* skills, we administered the Tower of London (Shallice, 1982), with available normative data based on a large sample and provides demographically adjusted indices for score, planning time, execution time, perseverations, rule breaks, and self-monitoring (Boccia et al., 2017). It requires participants to solve a series of problems by moving disks across pegs according to specific rules to compose specific configurations. *Cognitive flexibility* and *inhibition* were assessed with the Modified Card Sorting Test (MCST; Caffarra et al., 2004) which evaluates the ability to shift cognitive strategies in response to changing rules. This test has available Italian normative data (Caffarra et al., 2004), strong split-half reliability (0.92 ≤ *r*_*s*_ ≤ 0.95) (Kopp et al., 2021), while construct validity is supported by evidence showing that MCST scores are associated with executive-function measures and discriminate between clinical and control groups (Branco et al., 2021). This dimension was measured also by the TMT B-A (Reitan, 1958), which isolates executive control by measuring the additional cognitive demands imposed by task-switching. To further examine *inhibition* and *sensitivity to interference*, we administered the Listening Span Test (Borella et al., 2008), which required participants to process and recall information while simultaneously engaging in a secondary listening task; this test, used in its Italian adaptation, is grounded in the broader sentence/listening span literature, which has shown good split-half reliability for listening span measures (*r* = 0.86; Salthouse & Babcock, 1991) and adequate psychometric support for sentence-span tasks, including significant test–retest reliability (*r* = 0.73 −0.76) and convergence on a common working-memory factor (λ = 0.810; Waters & Caplan, 2003). As an additional index, the third part of SCWT (Stroop, 1935) was also administered, measuring the ability to suppress automatic responses by requiring participants to name the ink color of incongruent color-word pairs (Stroop CWT gives us a measure of time, while CWI provides the number of correct items).

#### Theory of Mind Measurement

To evaluate participants’ ability to understand and infer others’ mental states, before and after the training we administered a set of tasks assessing different components of Theory of Mind. As with the cognitive assessment, administration and scoring procedures for the ToM tasks were conducted by the same person.

*Affective ToM* was assessed via the Reading the Mind in the Eyes test (RMEt – revised; Baron-Cohen et al., 2001). The task measures social sensitivity and the ability to recognize emotions and mental states from subtle facial cues. Participants were shown black-and-white images depicting only the eye region of different faces and were asked to select the adjective that best described the mental state conveyed in each image. The RMEt has shown construct-validity support in the original study and, in the Italian validation, acceptable internal consistency (*α* = 0.605; maximal weighted reliability = 0.719) and good test–retest reliability (ICC = 0.833, 95% CI [0.745, 0.902]; Vellante et al., 2013).

*Cognitive* *ToM* was assessed via the Strange Stories task (SS; Happé, 1994). Participants listened to short stories and answered questions assessing their comprehension. The task is composed of control stories, aimed at evaluating the ability to understand explicit information and draw logical inferences, and mentalistic stories, requiring participants to infer the mental states and intentions of the characters; this task has shown psychometric support, with Cronbach’s *α* = 0.73 (Hayward & Homer, 2017) and construct validity is supported by latent-variable evidence on ToM factor (*ϕ* = 0.66; *p* <.001), showing good test–retest reliability (*ϕ* = 0.83, *p* <.001; Devine & Hughes, 2016). Additionally, the Second-order false belief task (Baron-Cohen, 1989; Perner & Wimmer, 1985) was used to examine the capacity to reason about others’ beliefs regarding third-party mental states; advanced false-belief tasks reported acceptable internal consistency (*α* = 0.66) and 4–6-week test–retest reliability (*r* = 0.66; Hughes et al., 2000). In this task, participants listened to a short story and answered questions probing their understanding of how one character perceives another character’s beliefs, thus assessing higher-order mental state attributions.

### Statistical Analysis

All statistical analyses were conducted on raw scores using parametric tests, as data normality was verified through the Shapiro-Wilk test (*p* >.05). Analyses were performed using SPSS (v29).

Repeated-measures ANOVAs (rmANOVAs) were performed to compare participants’ pragmatic performance at baseline (T0), post-training (T1), and at the three-month follow-up (T2). These analyses were conducted separately for the global ABaCo score, the composite production and comprehension scores, and the ABaCo scales. Multivariate effects were assessed using Pillai’s trace (*V*). The assumptions of sphericity, and homogeneity of variances were verified using Mauchly’s test and Levene’s test. When a significant main effect of the assessment time was found, Bonferroni-corrected post-hoc comparisons were performed. Effect sizes were reported using partial eta squared (*η*^*2*^_*p*_), with values of 0.01, 0.06, and 0.14 interpreted as small, medium, and large effects, respectively (Richardson, 2011). To assess the magnitude of the effects observed, a post-hoc calculation of the statistical power was performed.

To investigate whether cognitive and ToM performance changed from pre- to post-training, a series of paired t-tests was conducted to compare performance before (T0) and after the training (T1). Additionally, paired t-tests were used to assess changes in quality of life from pre- to post-training. Bonferroni corrections were applied to account for multiple comparisons and reduce the risk of Type I errors. For *t*-tests, effect sizes were reported using Cohen’s *d*, with 0.20, 0.50, and 0.80 indicating small, medium, and large effects, respectively (Cohen, 1988).

*Statistical power* Statistical power was calculated with G*Power 3.1 (Faul et al., 2007). For a repeated measures design with three measurement points, an alpha level of 0.05, and a sample size of 24 participants, we estimated the minimum effect size required to achieve 95% of power. Based on this configuration, an effect size of *f* = 0.34 was sufficient to reach the desired power. Using the standard conversion formula *η*^*2*^_*p*_ = *f²*/(*f²* + 1), this corresponds to a partial eta squared of approximately 0.10.

## Results

### Pragmatic Performance

**Fig. 4:**
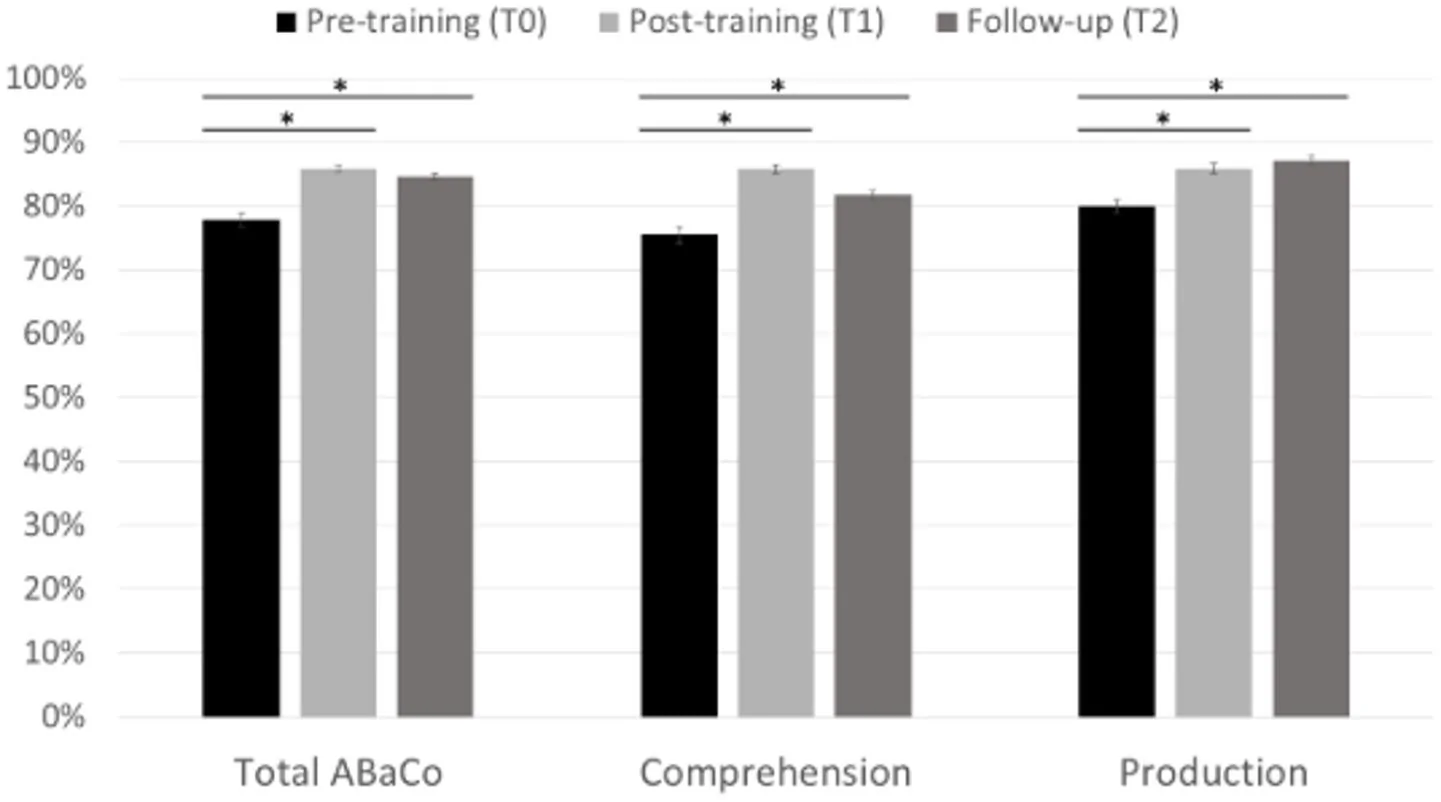
Mean scores (expressed in percentages by multiplying raw scores for 100) for the ABaCo total score and composite scores for pragmatic comprehension and production

**Fig. 5:**
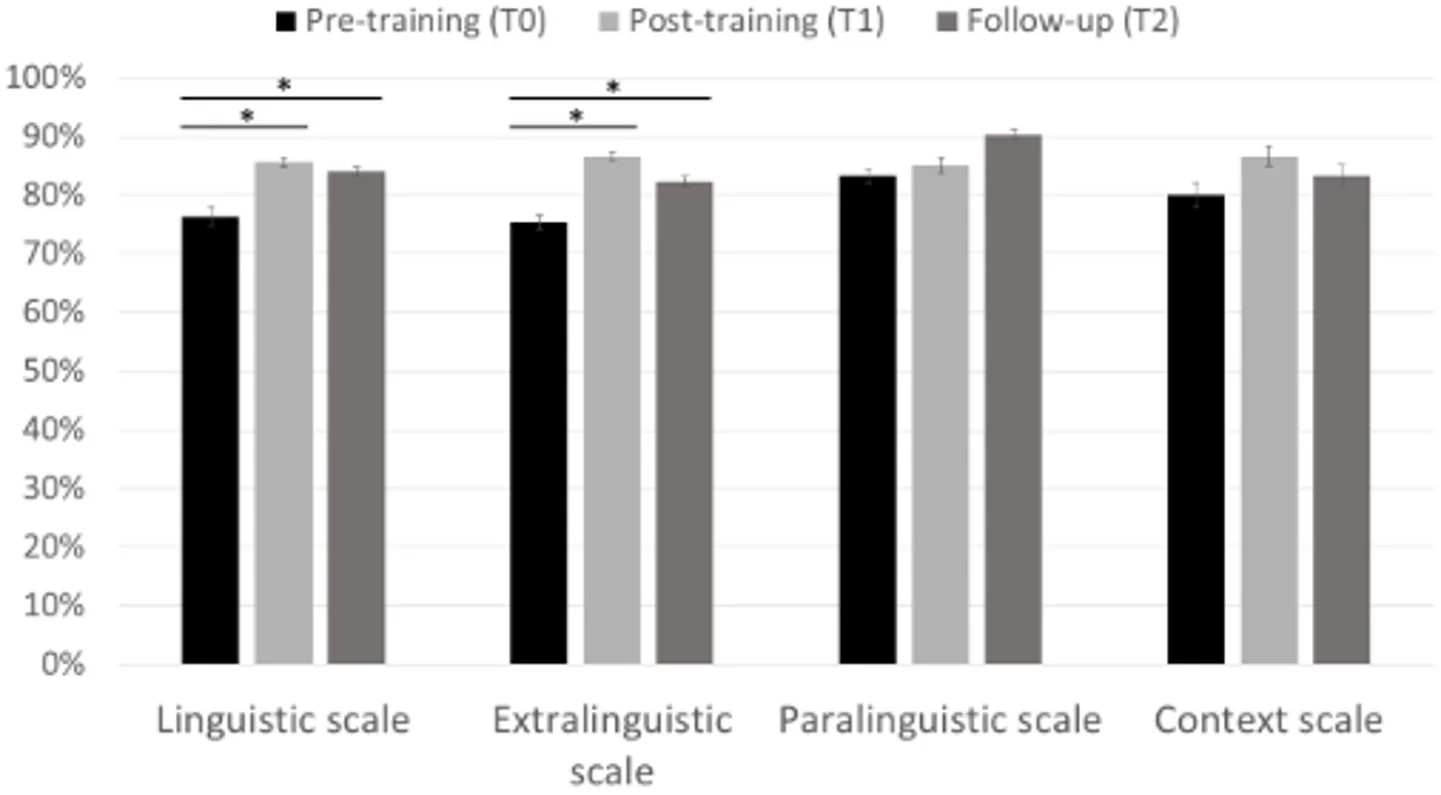
Mean performance scores of participants on the ABaCo scales, expressed in percentages by multiplying raw scores for 100

For pragmatic ability, we used a series of rmANOVAs to compare participants’ performance pre- (T0) and post-training (T1) and at a 3-month follow-up (T2) on ABaCo total score and scales. Descriptive statistics for the ABaCo scores and percentage changes across time points are reported in Table 2. Changes over time for the ABaCo total score and composite comprehension and production scores are shown in Fig. 4, while changes for the ABaCo scales are displayed in Fig. 5.

**Table 2:** Descriptive statistics (means and standard deviations) for ABaCo scores at pre-training (T0), post-training (T1) and at the 3-month follow-up (T2), with percentage changes across time points

Pragmatic measure	T0 Pre-training M ± SD	T1 Post-training M ± SD	T2 3-month follow-up M ± SD	% Change T0–T1	% Change T1–T2	% Change T0–T2
Total ABaCo	.78 ± .10	.86 ± .06	.85 ± .05	+ 10.31%	−1.50%	+ 8.65%
Comprehension	.76 ± 0.13	.86 ± .07	.82 ± .06	+ 13.72%	−4.69%	+ 8.38%
Production	.80 ± 0.10	.86 ± .08	.87 ± .07	+ 7.32%	+ 1.44%	+ 8.87%
Linguistic scale	.76 ± 0.15	.86 ± .08	.84 ± .08	+ 12.02%	−1.80%	+ 10.00%
Extralinguistic scale	.75 ± 0.12	.87 ± .06	.82 ± .08	+ 14.90%	−5.01%	+ 9.14%
Paralinguistic scale	.83 ± 0.12	.85 ± .14	.90 ± .09	+ 2.09%	+ 6.12%	+ 8.34%
Context scale	.80 ± 0.20	.87 ± .17	.83 ± .17	+ 8.34%	−3.85%	+ 4.16%

For ABaCo total score, the analysis revealed a significant effect of the assessment time (*F*(2, 22) = 12.97, *p* <.001; *η*^*2*^_*p*_ = 0.54) with a linear (*p* <.001) and quadratic trend (*p* <.001). Post-hoc Bonferroni comparisons revealed a significant improvement after training (T0 vs. T1: *p* <.001) that was stable over time (T1 vs. T2: *p* =.818) and still significant at the 3-month follow-up (T0 vs. T2: *p* =.002).

For comprehension ability, the rmANOVA revealed a significant effect of the assessment time (*F*(2, 22) = 8.54, *p* =.002; *η*^*2*^_*p*_ = 0.44) with a linear (*p* =.013) and quadratic trend (*p* <.001). Post-hoc Bonferroni comparisons showed that there was an improvement after training (T0 vs. T1: *p* =.001) which slightly decreased over time (T1 vs. T2: *p* =.049) but remained visible at follow-up (T0 vs. T2: *p* =.040).

Similarly, the rmANOVA on production ability revealed a significant effect of the assessment time (*F*(2, 22) = 7.28, *p* =.004; *η*^*2*^_*p*_ = 0.40) with a linear trend (*p* =.001). Post-hoc Bonferroni comparisons showed that there was a significant improvement after-training (T0 vs. T1: *p* =.027) that remained stable over time (T1 vs. T2: *p* = 1.000), showing a long-lasting improvement (T0 vs. T2: *p* =.004).

As for the linguistic scale, the rmANOVA revealed a significant effect of the assessment time (*F*(2, 22) = 6.80, *p* =.005; *η*^*2*^_*p*_ = 0.38) with a linear (*p* =.006) and quadratic trend (*p* =.005). Post-hoc Bonferroni comparisons revealed a significant improvement after training (T0 vs. T1: *p* =.003) that was stable over time (T1 vs. T2: *p* = 1.000) and still held at the 3-month follow-up (T0 vs. T2: *p* =.017).

For the extralinguistic scale, the rmANOVA revealed a significant effect of the assessment time (*F*(2, 22) = 10.63, *p* <.001; *η*^*2*^_*p*_ = 0.49) with a linear (*p* =.005) and quadratic trend (*p* <.001). Post-hoc Bonferroni comparisons revealed a significant improvement after training (T0 vs. T1: *p* <.001) that was stable over time (T1 vs. T2: *p* =.096) and still detectable at the follow-up (T0 vs. T2: *p* =.014).

By contrast, the rmANOVA revealed no significant effect of the assessment time for the paralinguistic scale (*F*(2, 22) = 3.19, *p* =.061; *η*^*2*^_*p*_ = 0.23) nor for the context scale (*F*(2, 22) = 1.27, *p* =.299; *η*^*2*^_*p*_ = 0.10), analyzed separately. However, a trend toward improvement over time can be observed in Fig. 5, particularly for the paralinguistic scale. In line with this pattern, polynomial contrast analyses revealed a significant linear trend for the paralinguistic scale (*p* =.026), suggesting a gradual increase in performance across sessions.

### Cognitive and Theory of Mind Assessment

To assess whether the training caused any changes in cognitive and social cognition functioning, we performed a series of paired sample *t*-tests, corrected with Bonferroni to account for multiple comparisons. Specifically, corrections were applied separately for the sets of cognitive tests (15 comparisons) and the ToM tasks (4 comparisons). The results are shown in Table 3. No significant changes were detected after training for cognitive abilities (*p* >.05) nor in the ToM tasks (*p* >.05).

**Table 3:** Performance scores pre- and post-training at the cognitive and ToM assessment

	T0 – Pre-training	T1 – Post-training	t	*p*	*p* ^a^	d
	M ± SD	M ± SD				
*Attention*						
TMT – A	43.73 ± 15.61	45.25 ± 17.06	−0.60	.553	1	.12
TMT – B	106.34 ± 81.54	104.08 ± 105.98	0.35	.729	1	.07
*Memory*						
Digit span – forward	7.33 ± 1.74	7.54 ± 2.09	−0.51	.615	1	.10
Immediate and deferred recall	12.72 ± 3.01	13.10 ± 3.27	−0.65	.520	1	.13
*Processing speed*						
Stroop WT	53.88 ± 15.01	49.51 ± 8.07	1.36	.184	1	.28
Stroop WI	60.33 ± 11.48	62.29 ± 10.72	−0.84	.394	1	.17
Stroop CT	87.67 ± 31.25	81.54 ± 26.63	1.48	.113	1	.30
Stroop CI	38.63 ± 9.66	41.83 ± 9.48	−2.82	.008	0.15	.58
*Executive functions*						
Digit span – backward	6.17 ± 1.90	6.54 ± 2.32	−1.18	.250	1	.24
TMT – B-A	62.61 ± 68.46	58.83 ± 93.57	0.57	.578	1	.12
Listening span	0.26 ± 0.38	0.25 ± 0.48	0.31	.041	1	.06
WCST	39.71 ± 6.12	40.19 ± 4.95	−0.37	.719	1	.08
Stroop CWT	214.58 ± 178.35	181.46 ± 95.64	1.76	.091	1	.36
Stroop CWI	18.54 ± 6.04	20.63 ± 6.92	−2.53	.019	.285	.52
Tower of London	23.08 ± 4.28	24.08 ± 4.27	−1.17	.253	1	.24
*Theory of mind*						
Mentalistic SS	9.92 ± 2.39	9.92 ± 1.84	0.00	1	1	.00
Control SS	6.58 ± 1.35	6.75 ± 1.51	0.58	.567	1	.12
II order stories	1.79 ± 0.42	1.67 ± 0.57	1.37	.163	.740	.28
RMEt	20.17 ± 4.13	21.33 ± 4.31	−1.44	.582	.652	.29

^a^*p*-values were adjusted using the Bonferroni correction for multiple comparisons

They were corrected separately for the sets of cognitive tests (15 comparisons) and the ToM tasks (4 comparisons)

## Discussion

The present preliminary study aimed to examine whether participation in the Advanced-CPT, a novel training program targeting the inferential dimension of communication and different means of expression, was associated with improvements in pragmatic ability in healthy aging. This topic is relevant from both research and clinical perspectives , as well as in a broader societal view. The world population, and the Italian one particularly, has recently undergone substantial population aging, which is supposed to continue and intensify in the coming decades (European Commission, 2024). Increased life expectancy leads to opportunities but also to several challenges that must become a priority for research and policies, as they may affect quality of life and increase social isolation. Research on pragmatic ability in aging has progressed slowly in recent years, also due to the lack of tools capable of accurately detecting weaknesses and strengths and to counteract possible decline. Therefore, providing tools for the assessment and the intervention in those areas particularly affected by the aging process, as is the case for pragmatic ability, provides a concrete response to demographic changes.

The preliminary data of the present study indicate that the newly developed Advanced-CPT appers to improve overall pragmatic ability: we observed a robust and statistically significant 10.31% improvement from pre- to post-training, which remained stable at follow-up with a mean increase from baseline of 8.65%. This pattern is consistent with a beneficial change in general pragmatic competence, with maintenance of gains over time. The great magnitude of the effect emphasizes the practical relevance of this change. A similar pattern of improvement holds for the composite pragmatic production and comprehension abilities. Both domains showed significant improvements after the intervention. While comprehension shows a slight decline from post-training to follow-up, the improvement from pre-training to follow-up remains statistically detectable. Consistent with the total score, production scores were fully maintained at follow-up, with no decline observed. In line with the results of Bambini et al. (2020) who reported, following their PRAGMACOM intervention, greater improvements in comprehension – measured by the Physical and Mental Metaphors task – than in production – measured by the propensity for off-topic speech during a semi-structured interview – our participants showed the same pattern after the training, with a mean increase of 13.72% in comprehension scores and of 7.32% in production scores. However, follow-up results with the Advanced-CPT showed comparable gains in both the expressive and receptive components of pragmatic communication. This balanced long-term improvement suggests that the Advanced-CPT provides a comprehensive stimulation of pragmatic skill, potentially due to its design or the specific training material used.

A closer look at the ABaCo scales revealed improvements on the linguistic and extralinguistic scales, which were also maintained at follow-up. These results suggest that participants became more adept at understanding and producing both verbal content and non-verbal cues such as gestures and facial expressions. Notably, these findings offer an important counterpoint to the evidence reported by Hilviu et al. (2022), in which the extralinguistic scale was found to be the means of expression most affected by age-related decline. Our results suggest that such deterioration may be reversible through targeted intervention, with participants showing an average improvement of 14.90% post-training and retaining a 9.14% gain at follow-up relative to baseline. Interestingly, improvements were also observed in the linguistic scale, although this expressive mean did not differ significantly between groups in the same study, suggesting that this ability, while relatively preserved, may still benefit from structured training. For this scale, a mean percentage increase of 12.02% was obtained post-training and a 10% increase from baseline was still detectable at follow-up. Although changes in the paralinguistic and contextual scales did not reach statistical significance, the direction of the raw scores and percentage changes align with the expected trend, indicating a potential positive effect with a long-term mean increase of approximately 8% for paralinguistic cues and of 4% for contextual abilities. These findings support the idea that paralinguistic and contextual abilities, previously identified as particularly vulnerable to age-related deterioration (Hilviu et al., 2022), may still respond to intervention, albeit requiring a larger sample size to detect significant changes. Therefore, further studies with larger cohorts of participants and a control group are needed to confirm these preliminary qualitative observations and to better understand the potential for improvement in these domains. It is important to note that the use of equivalent forms of ABaCo pre- and post-training reduces the likelihood that the improvements from T0 to T1 were merely due to practice effects, strengthening the interpretation that the observed gains reflect genuine learning and transfer. However, it should be acknowledged that despite the use of the equivalent forms of ABaCo, designed to reduce learning effects in multiple assessment times, the T0 and T2 assessments both employed the equivalent form A, and a residual practice effect at follow-up cannot be entirely excluded. That said, as previously argued by Bambini and colleagues (2020), who administered the same assessment tasks at both T0 and T1 to evaluate PRAGMACOM’s efficacy, three features of this preliminary work collectively mitigate this concern: the substantial temporal interval between assessments, as approximately five months elapsed between baseline and follow-up evaluations, and two additional characteristics of ABaCo itself. Apart from four paralinguistic items, ABaCo does not require participants to select from pre-coded response options, which would be more susceptible to memorization, but instead requires them to actively articulate their responses. Moreover, no feedback is provided on performance at any time point, limiting participants’ ability to learn from the previous administration.

Interestingly, as expected, no significant pre-post changes emerged in cognitive functioning or ToM performance, as assessed by standardized tasks used as control measures. While a few tasks showed medium effect sizes and lower *p*-values (e.g., processing speed and inhibition), no result remained significant after correction for multiple comparisons. It is noteworthy that, as equivalent forms are not available for most of the cognitive and ToM tasks, identical test versions were used before and after training; this might have led to learning effects and may partly explain the numerical differences observed, which still did not reach statistical significance. These results suggest that the improvements in pragmatic performance are not merely byproducts of broader cognitive or social-cognitive changes, but rather reflect specific gains in communicative competence attributable to the content and structure of the training itself.

These preliminary results provide initial support for the potential usefulness of an intervention focused on the inferential dimension of communication and on the integration of multiple expressive modalities for promoting pragmatic performance in healthy aging. Such inferential and multi-dimensional approach has proven beneficial not only in healthy aging populations but also in a variety of clinical conditions. In its original version, indeed, the CPT has proven effective with individuals with traumatic brain injury (Bosco et al., 2018a, b, c; Gabbatore et al., 2015; Parola et al., 2019; Sacco et al., 2016), schizophrenia (Bosco et al., 2016; Gabbatore et al., 2017) and in autistic adolescents (Gabbatore et al., 2022). Across these populations, the CPT has demonstrated its capacity to specifically focus on weaknesses and strengths characterizing each pragmatic profile and lead participants to an improvement and an enhancement of specific components of both pragmatic comprehension and production.

Compared to the original CPT, an innovation of the Advanced-CPT was the introduction of materials and activities focusing on intergenerational communication. The reason for this was to contribute to the reduction of social isolation: a sense of exclusion in aging may depend on the difficulty of dealing with new media, means and modalities of communication, which are rapidly evolving and lead to misunderstanding that may widen the gap between older adults and members of younger generations. The inclusion of specific activities centered on intergenerational communication allowed participants to practice their ability to draw inferences from new idiomatic expressions, metaphors and colloquial phrases commonly used in contemporary language. Moreover, specific exercises focus on communicative appropriateness when using new digital communication tools such as email, instant messaging and social media to help participants navigate among the options and choose the best way to communicate depending on the context. To the best of our knowledge this is the first time that such aspects have been introduced in a training program for pragmatics in old age; that makes the Advanced-CPT particularly valuable for promoting more effective and confident interactions in elderly individuals, increasing the sense of efficacy and autonomy. It is also worth noting that in the present study there was only one dropout, which occurred for medical reasons. Among the possible contributing factors, we may hypothesize a role of several innovative features of the program, including its structure and activities, as well as the small group format of 5 to 6 participants which ensures that each individual has sufficient space to practice and exchange during every session, while also facilitating the scheduling of appointments around participants’ personal commitments, supporting its ecological validity and feasibility as an intervention. However, the characteristics of the sample should also be considered. Participants were characterized by a relatively high mean educational level (*M* = 13.79 years), and recruitment partly relied on a lifelong-learning institution, public-engagement activities, and word-of-mouth channels. These features may reflect comparatively high cognitive reserve and motivation, which likely favored adherence and may also have contributed to the magnitude of the observed gains. Future studies should therefore involve larger and more heterogeneous samples, including participants with broader educational backgrounds and varying levels of motivation, to clarify the generalizability of these preliminary findings and disentangle program-related effects from sample-selection effects.

The overall preliminary results of the present study align with those of Bambini and colleagues (2020), who focused on language only proved that cognitive plasticity is still present in late adulthood and suggest that pragmatics, like other cognitive functions (e.g., memory and EFs) (Borella et al., 2010; Cavallini et al., 2015a, b; López-Martínez et al., 2011; Wang et al., 2011), and like ToM (Cavallini et al., 2015a, b), is adaptable and can benefit from targeted interventions. Here we extended such observations in a multimodal perspective, highlighting that changes may occur not only for specific linguistic skills, but also in the use of gestures, i.e., extralinguistic, in the connotation of the speech via tone and rhythm, i.e., paralinguistic, and in the adherence to the context in which the communicative interaction takes place. Taken together, these findings are consistent with the possibility that guided and structured changes in pragmatic activity and engagement are associated with corresponding improvements in pragmatic performance. As suggested by Bambini and colleagues (2020), future research should include neuroimaging techniques that could provide valuable insights into the neural responses elicited by the Advanced-CPT and contribute to a deeper understanding of the mechanisms underlying pragmatic plasticity in healthy aging.

The results of this study should be interpreted within the background of its limitations. Despite an achieved statistical power of 95% thanks to the large effect sizes observed, it should be noted that the relatively small sample size may limit the generalizability of the results. Smaller samples are more susceptible to variability; future research should therefore aim to replicate these findings in a larger sample. In addition to that, in the absence of a control group, the present study falls within a quasi-experimental design, and causal inferences about the effectiveness of Advanced-CPT are necessarily limited. Even if the addition of a control group is recommended (see for example Bambini et al., 2020, 2022), single-group designs are not uncommon in the early stages of training development (Bosco et al., 2016; Gabbatore et al., 2015, 2022), and the choice to begin evaluating Advanced-CPT in this way was also driven by practical considerations: given the substantial time commitment required of both participants and experimenters for assessment and training, this preliminary study allows us to optimize resources in future investigations and to refine the protocol before scaling up to a larger, controlled trial, before the intervention has been adequately piloted. We are aware of the limitations inherent in single-group designs (see Paulus et al., 2014 for a review), and future research should aim to replicate these findings using a randomized controlled design, including a control group not participating in the training, to provide a more robust assessment of the effectiveness of Advanced-CPT and to rule out alternative explanations such as spontaneous improvement or confounding effects.

## Supplementary Information

Below is the link to the electronic supplementary material.

Supplementary Material 1

## Data Availability

The datasets generated during and/or analyzed during the current study are not publicly available for privacy or ethical restrictions; however, data will be available from the corresponding author (giorgia.vegna@unito.it) on reasonable request.
