# Supplementary material for: Enhancing Pragmatic Communication in Healthy Aging: A Preliminary Investigation of the Long-Term Effects of the Advanced Cognitive Pragmatic Treatment
Source: J Psycholinguist Res. 2026 Aug 21;55(5):110. doi: 10.1007/s10936-026-10291-4 (PMC13498483; doi:10.1007/s10936-026-10291-4)
Supplement: Supplementary file 1 [file 10936_2026_10291_MOESM1_ESM.docx]

**Supplementary Material**

**Full description of the Advanced CPT**

|  | 9 weekly sessions,  About 90 min/session with a 10-minute break |
| --- | --- |
|  | Groups of 5/6 participants |
|  | All the sessions are videorecorded to enable:  - working on self-awareness pre/post training  – highlighting improvements  – creating a “best of” videoclip at the end of the training |
|  | The Advanced CPT addresses all the expressive modalities, not only language |
|  | The activities focuses on both pragmatic comprehension and production |
|  | Each session starts with a recap of what has been previously done  And ends with conclusions and homework (to keep working at home) |

| **Session 1 - Initial Presentation and Awareness** | |
| --- | --- |
| *Participants are invited to reflect on their motivations and expectations for the program. The session introduces communication as a collaborative process rather than a simple transfer of information, emphasizing the integration of linguistic and extralinguistic elements.* | |
| ***Example of activity*** | |
| *Aim of the task:* Raising awareness of the discrepancy between proper communication and merely one-sided transmission of information.   One participant – the *describer*- describes a given picture to all the others –*drawers.*  Phase 1: The *drawers* can’t see the picture and can only draw on the basis of the instructions provided by the *describer*, without asking questions.  Phase 2: The *drawers* can interact with the *describer* to ask questions and clarifications.  The outcome is commented on at the end of the activity based on the aim of the task. | Obiettivo: Aumentare la consapevolezza della discrepanza tra una comunicazione efficace e una mera trasmissione unilaterale di informazioni.  Un partecipante, il *descrittore*, descrive un’immagine a tutti gli altri, i *disegnatori*.  Fase 1: I *disegnatori* non possono vedere l’immagine e possono disegnare solo sulla base delle istruzioni fornite dal descrittore, senza fare domande.  Fase 2: i *disegnatori* possono interagire con il descrittore per fare domande e chiedere chiarimenti.  Alla fine dell’attività, il risultato viene commentato sulla base dell’obiettivo del compito. |
| **Session 2 - Fundamental Communicative Skills** | |
| *Focus on communicative intent and the importance of interpreting meaning beyond verbal content. Participants learn to consider extralinguistic (i.e., gestures, posture, tone of voice) and contextual cues (shared background knowledge) for deeper understanding.* | |
| ***Example of activity - Production*** | |
| *Aim of the task*: practicing communicative ability in a conscious and controlled setting, paying attention to the elements of communicative interactions    The participants work in pairs and are given a prototypical situation in which each of them plays a certain predefined role:  Example: Doctor's office. (  Participant A*:* You are waiting to meet your doctor to report some symptoms that are worrying you; you want the doctor to give you a prescription for an expensive and invasive test.  Participant B*:* You play the role of the young doctor working at this clinic. When you hear about the symptoms described, you understand that it is not a very serious condition and that it can be easily treated by taking some regular medications | *Obiettivo*: Esercitare l’abilità comunicativa in un contesto consapevole e controllato, prestando attenzione agli elementi delle interazioni comunicative.  I partecipanti lavorano in coppia e viene loro proposta una situazione prototipica in cui ciascuno interpreta un ruolo prestabilito.  Esempio: studio medico.  *Partecipante A:* Stai aspettando di incontrare il tuo medico per riferire alcuni sintomi che ti preoccupano; vuoi che il medico ti prescriva un esame costoso e invasivo.  *Partecipante* B: Interpreti il ruolo di un giovane medico che lavora in questo ambulatorio. Quando senti i sintomi descritti, capisci che non si tratta di una condizione molto grave e che può essere facilmente trattata con farmacoterapia comune. |
| **Session 3 - Extralinguistic Skill** | |
| *Highlights the role of nonverbal communication, such as facial expressions and gestures, in conveying meaning. Participants develop awareness of how nonverbal signals provide critical information about a speaker’s emotions and communicative intent.* | |
| ***Example of activity - comprehension*** | |
| *Aim of the task*: highlighting in communication language is not the only component, as often gestures and facial expression provide significant meanings.  Participants are shown brief videoclips, from movies from movies or TV series featuring characters engaged in a communicative interaction. The clips is presented without sound. They are asked to observe and notice actors’ facial expressions and gestures, and guess their intentions and emotions. | *Obiettivo*: Evidenziare come, nella comunicazione, il linguaggio non sia l’unica componente, poiché spesso gesti ed espressioni facciali veicolano significati importanti.  Ai partecipanti sono mostrati brevi videoclip tratti da film/serie TV, in cui i personaggi sono impegnati in un’interazione comunicativa. I clip vengono presentati senza audio. Viene quindi chiesto notare e codificare espressioni facciali e gesti degli attori, e indovinarne intenzioni e emozioni. |
| **Session 4 - Paralinguistic Skill** | |
| *Explores how tone, intonation, and prosody shape communication. Participants reflect on the ways these elements contribute to meaning, emotion, and social interpretation.* | |
| ***Example of activity - comprehension*** | |
| *Aim of the task:* Focusing on the emotional connotation that the tone of voice can convey, beyond language.   Participants are asked to read out short sentences from a list, using a specific emotional intonation (e.g., sad, happy, anxious, etc.). Each participant (reader) takes it in turn to read out a sentence, while the others are asked to interpret the emotion expressed by the reader’s tone of voice. The intonation of the reader is then compared with a recording of the same sentence read by an actor with the correct intonation. A brief discussion follows, based on the aim of the task. | *Obiettivo*: Focalizzare attenzione sulla connotazione emotiva che il tono di voce può trasmettere, al di là del linguaggio.  Ai partecipanti viene chiesto di leggere ad alta voce brevi frasi tratte da una lista, usando una specifica intonazione emotiva, ad esempio triste, felice, ansiosa, ecc. A turno, ogni partecipante (*lettore*) legge una frase, mentre agli altri viene chiesto di interpretare l’emozione espressa attraverso il tono di voce del *lettore*.  L’intonazione del *lettore* viene poi confrontata con una registrazione della stessa frase letta da un attore, con l’intonazione corretta. Segue una breve discussione basata sull’obiettivo del compito. |
| **Session 5 - Cognitive Flexibility in Communication** | |
| *Emphasizes the ability to adapt communication based on the listener’s needs and feedback. Participants practice producing contributions that are clear, relevant, and appropriately detailed.* | |
| ***Example of activity*** | |
| *Aim of the task:* highlighting the importance of adapting the content to the interlocutor’s knowledge and needs, for an effective communication.  Two participants (*director* and *directee*) are provided two maps, containing different information (with/without street names; with/without graphical elements). The *director* verbally guides the directee along a route toward a hidden destination, not revealing the destination but providing progressive infos; the directee follows the instructions and provides feedback. | *Obiettivo*: Evidenziare l’importanza di adattare il contenuto alle conoscenze e ai bisogni dell’interlocutore, per una comunicazione efficace.  A due partecipanti (*guida* e *destinatario*) vengono fornite due mappe contenenti informazioni diverse, i.e., con o senza nomi delle strade e con o senza elementi grafici. La *guida* orienta verbalmente il *destinatario* lungo un percorso verso una destinazione nascosta, senza rivelarla ma fornendo informazioni progressive; il *destinatario* segue le istruzioni e fornisce feedback. |
| **Session 6 - Clarity and Effectiveness in Expression** | |
| *Reinforces the need to adjust communication based on the interlocutor and context. Participants work on structuring messages that are concise, relevant, and easily understood.* | |
| ***Example of activity - comprehension*** | |
| *Aim of the task:* promoting a reflection on the proper use of linguistic referents during a conversation, to avoid confusion.  Participants are given a short written first-person narrative in which several characters are introduced over time. Characters are not identified by name, but only through pronouns (he/she, that/this one), making it unclear who the author is referring to.  Participants are asked to read the story aloud and, through guided questions, discuss the intelligibility of the text. | Obiettivo: Promuovere una riflessione sull’uso appropriato dei referenti linguistici durante una conversazione, per evitare confusione.  Ai partecipanti viene fornito un breve racconto scritto in prima persona, nel quale vengono introdotti, uno dopo l’altro, diversi personaggi. I personaggi non sono identificati per nome, ma solo tramite pronomi (lui/lei, quello/questa), in modo da rendere poco chiaro a chi l’autore si stia riferendo.  Ai partecipanti viene chiesto di leggere la storia ad alta voce e, attraverso domande guidate, di commentare l’intelligibilità del testo. |
| **Session 7 – Narrative Skills** | |
| *Focuses on distinguishing between relevant and irrelevant information for effective storytelling. Participants practice summarizing key points while reducing verbosity.* | |
| ***Example of activity - comprehension*** | |
| *Aim of the task:* enhancing the ability to pay attention to and select the most relevant information and narrate them effectively.  One participant at a time watches a short video (e.g., advertisement) and is asked to describe the content, as clearly and effectively as possible, to an audience unfamiliar with the video. This is followed by a group reflection and discussion. | *Obiettivo*: Potenziare la capacità di prestare attenzione alle informazioni più rilevanti, selezionarle e narrarle in modo efficace.  Un partecipante alla volta guarda un breve video (cortometraggio/pubblicità). La richiesta è di dare una descrizione del contenuto nel modo più chiaro ed efficace possible, per un pubblico che non conosce il video. Seguono riflessione e discussione di gruppo. |
| **Session 8 – Social Appropriateness and Digital Communication** | |
| *Encourages reflection on how communication tools and language registers should align with social context and message content. Participants discuss how to adjust tone and style in different social and digital interactions.* | |
| ***Example of activity - production*** | |
| *Aim of the task:* reflect on the use of communication tools and registers most appropriate and consistent with the content and the social relationship among the speakers.  The participants work in pairs and are given a prototypical situation in which each of them plays a certain predefined role. Participants are told that the simulated situation does not involve face-to-face communication, but that the interaction will be mediated by a tool, such as a computer.  *Example:*  *Participant A.* You are planning to organize a dinner with old school friends. You want to gather as many contacts as possible to invite people, while keeping the planning quick and simple and receiving practical advice, such as where to hold the dinner and whom to invite. Therefore, you decide to contact *Participant B*, an old school friend you have not been in touch with for a very long time. You want to get in contact with *B* for the get together and want to make sure to get an answer as soon as possible.  *Participant B. You are Participant A’s old school friend, even* though over the years you have somewhat lost touch. However, you have stayed in contact with many of your former classmates, and you really enjoy reunions. | *Obiettivo*: riflettere sull’uso degli strumenti e dei registri comunicativi più appropriati e coerenti con il contenuto e con la relazione sociale tra gli interlocutori.  I partecipanti lavorano in coppia su una situazione prototipica in cui ciascuno interpreta un ruolo prestabilito. Viene specificato che la situazione simulata non prevede una comunicazione faccia a faccia, ma che l’interazione sarà mediata da uno strumento, come per esempio il computer.  *Esempio*: *Partecipante A*. Stai organizzando una cena con vecchi amici di scuola. Vuoi raccogliere il maggior numero possibile di contatti per invitare le persone, mantenendo però l’organizzazione rapida e semplice e ricevendo consigli pratici, ad esempio su dove tenere la cena e chi invitare. Per questo decidi di contattare il *Partecipante B*, un vecchio amico di scuola con cui non sei in contatto da molto tempo. Vuoi metterti in contatto con *B* per organizzare l’incontro e vuoi assicurarti di ricevere una risposta il prima possibile.  *Partecipante B****.*** Sei un vecchio amico di scuola di *A*, anche se nel corso degli anni vi siete persi di vista e non vi sentite da molto tempo. Tuttavia, sei rimasto in contatto con molti ex compagni di classe e ti piacciono molto le riunioni tra vecchi amici. |
| **Session 9 – Final Session and Self-Reflection** | |
| *Participants review key concepts from the program and analyze personal improvements using video excerpts from previous sessions. The course concludes with a discussion on progress, final reflections, and participant feedback.* | |
| ***Example of activity*** | |
| The conductors draw together the overall work completed and review what was covered during the sessions.  A round-table discussion is held so that each participant can provide feedback on:  - usefulness of the sessions;  - highlights and weaknesses  - most improved aspects of communication;  - aspects of communication they still feel weak in;  …  Each participant’s self-presentation video from the first session is shown, followed by selected clips from the training highlighting improvements in the skills addressed.  A discussion follows on the content of the videos and the improvements observed. | I conduttori tirano le fila del lavoro svolto e ripercorrono quanto è stato trattato nel corso delle sessioni.  Una discussione a giro di tavolo coinvolge e richiede feedback a ciascun partecipante su:  - l’utilità degli incontri;  - i punti di forza e di debolezza;  - gli aspetti della comunicazione in cui ha registrato i maggiori miglioramenti;  - gli aspetti della comunicazione in cui si sente ancora debole;  …  Viene mostrato il video di presentazione di sé di ciascun partecipante realizzato durante il primo incontro, seguito da clip selezionate che evidenziano i miglioramenti nelle abilità trattate lungo il training. Segue una discussione sul contenuto dei video e sui miglioramenti osservati. |
